# Supplementary material for: Empirical methods for controlling false positives and estimating confidence in ChIP-Seq peaks
Source: BMC Bioinformatics. 2008 Dec 5;9:523. doi: 10.1186/1471-2105-9-523 (PMC2628906; doi:10.1186/1471-2105-9-523)
Supplement: Additional File 1 — A variety of html documents from the USeq web site detailing the available applications, their best usage, output file type descriptions, command line menus, etc. [file 1471-2105-9-523-S1.zip › USeqUserGuides/JavaDoc.zip!/JavaDoc/util/bio/parsers/class-use/UCSCGeneLine.html]

Uses of Class util.bio.parsers.UCSCGeneLine


|  |  |  |
| --- | --- | --- |
| **Overview** \| **Package** \| **Class** \| **Use** \| **Tree** \| **Deprecated** \| **Index** \| **Help** | |  |
| PREV   NEXT | **FRAMES**    **NO FRAMES**     **All Classes** |  |


---


## **Uses of Class util.bio.parsers.UCSCGeneLine**

| Packages that use UCSCGeneLine | |
| --- | --- |
| **trans.main** | Principle classes and scripts used in TiMAT2 analysis. |
| **trans.misc** | Rough working scripts. |
| **trans.roc** | For manipulating the BAC dmel spike in data and generating ROC curves. |
| **util.bio.annotation** | For representing gene annotation. |
| **util.bio.parsers** | Parsers and extractors, fasta, quality, text, gff.... |

Uses of UCSCGeneLine in trans.main

| Methods in trans.main that return UCSCGeneLine | |
| --- | --- |
| `UCSCGeneLine` | `ScoredGene.getGene()` |

Constructors in trans.main with parameters of type UCSCGeneLine

`ScoredGene(UCSCGeneLine geneReference,
float[][] treatmentValues,
float[][] controlValues)`

Uses of UCSCGeneLine in trans.misc

| Methods in trans.misc with parameters of type UCSCGeneLine | |
| --- | --- |
| `boolean` | `CalculateGeneExtensions.intersects(UCSCGeneLine first, int start, int end)`             Assumes coordinates are inclusive. |

Uses of UCSCGeneLine in trans.roc

| Methods in trans.roc with parameters of type UCSCGeneLine | |
| --- | --- |
| `void` | `ScoreParsedBars.calculateMedians(UCSCGeneLine[] lines)`             Calculates the median intensity for the oligos that lie under a gene. |

Uses of UCSCGeneLine in util.bio.annotation

| Methods in util.bio.annotation with parameters of type UCSCGeneLine | |
| --- | --- |
| `java.lang.String[]` | `ExportIntronicRegions.fetchIntrons(boolean[] mask, UCSCGeneLine[] genes)` |
| `boolean[]` | `ExportIntronicRegions.makeExonMask(UCSCGeneLine[] genes)` |

Uses of UCSCGeneLine in util.bio.parsers

| Methods in util.bio.parsers that return UCSCGeneLine | |
| --- | --- |
| `UCSCGeneLine[]` | `UCSCGeneModelTableReader.getGeneLines()` |

| Methods in util.bio.parsers with parameters of type UCSCGeneLine | |
| --- | --- |
| `void` | `UCSCGeneModelTableReader.setGeneLines(UCSCGeneLine[] geneLines)` |

---


|  |  |  |
| --- | --- | --- |
| **Overview** \| **Package** \| **Class** \| **Use** \| **Tree** \| **Deprecated** \| **Index** \| **Help** | |  |
| PREV   NEXT | **FRAMES**    **NO FRAMES**     **All Classes** |  |


---
